# Three-dimensional electron diffraction on clinkers: the belite α′_*H*_ incommensurate modulated structure

**DOI:** 10.1107/S205252062400146X

**Published:** 2024-03-06

**Authors:** Sergi Plana-Ruiz, Emilia Götz, Thomas Neumann, Peter Schwesig, Ute Kolb

**Affiliations:** a Servei de Recursos Científics i Tècnics, Universitat Rovira i Virgili, Avinguda Països Catalans 26, Tarragona, Catalonia 43007, Spain; b Institut für Angewandte Geowissenschaften, Technische Universität Darmstadt, Petersenstrasse 23, Darmstadt, Hessen 64287, Germany; c Schwenk Zement GmbH & Co. Kg, Laudenbacher Weg 5, Karlstadt, Bavaria 97753, Germany; d Master Builders Solutions Deutschland GmbH, Dr.-Albert-Frank-Strasse 32, Trostberg, Bavaria 83308, Germany; e Institut für Anorganische Chemie und Analytische Chemie, Johannes Gutenberg University of Mainz, Duesbergweg 10-14, Mainz, Rheinland-Pfalz 55128, Germany; University of Antwerp, Belgium

**Keywords:** three-dimensional electron diffraction, 3D ED, electron microscopy, modulated structures, cement

## Abstract

Traditional X-ray methods are extensively applied to commercial cement samples in order to determine their physical and chemical properties. Powder patterns are routinely used to quantify the composition of these phase mixtures, but structure determination becomes difficult because of reflection overlapping caused by the high number of different crystal structures. The fast-growing 3D electron diffraction technique and its related automated acquisition protocols arise as a potentially very interesting tool for the cement industry, since they enable the fast and systematic acquisition of diffraction data from individual particles. In this context, electron diffraction has been used in the investigation of the different crystalline phases present in various commercial clinkers for cement. Automated data collection procedures and subsequent data processing have enabled the structural characterization of the different crystal structures from which the α′_*H*_ polymorph of Ca_2_SiO_4_ (belite) exhibited satellite reflections. Its average crystal structure has been known since 1971 and satellite reflections have been reported previously, yet the modulation was never fully described by means of the superspace formalism. Here, the incommensurately modulated structure is solved and refined using harmonic and crenel functions in the superspace group *Pnma*(α00)0*ss*, showing the potential of 3D electron diffraction for systematic crystallographic characterizations of cement. A full description of the different belite polymorphs is provided considering this modulated structure.

## Abstract 2

Three-dimensional electron diffraction is comprehensively used to characterize different specimens of commercial clinkers for cement. The crystallographic analysis has enabled the crystal structure determination of belite α′_*H*_ as an incommensurate modulated structure with **q** ≃ 0.376a* and superspace group *Pnma*(α00)0*ss*.

## 1. Introduction

Cement is a key material in our society as it is a strong building material that can withstand long periods of time. This results in cement being the largest manufactured product by mass on Earth. Combined with water and mineral aggregates it forms concrete, the second most used substance in the world due to its simple production from widely available materials, its easiness to place it and adjust it to complex geometries, and its high strength and density (Scrivener *et al.*, 2018 ▸). Nevertheless, one of the major concerns is the emission of CO_2_, which is mainly driven by decarbonatization (around 40% of produced CO_2_ comes from de-calcination of limestone). Its production dependence on the burning of fossil fuels contributes up to 8% of global anthropogenic carbon dioxide emissions, thus its proper characterization to foresee alternative components is needed to reduce its impact (Mahasenan *et al.*, 2003 ▸; Olivier *et al.*, 2016 ▸).

**Figure 1:**
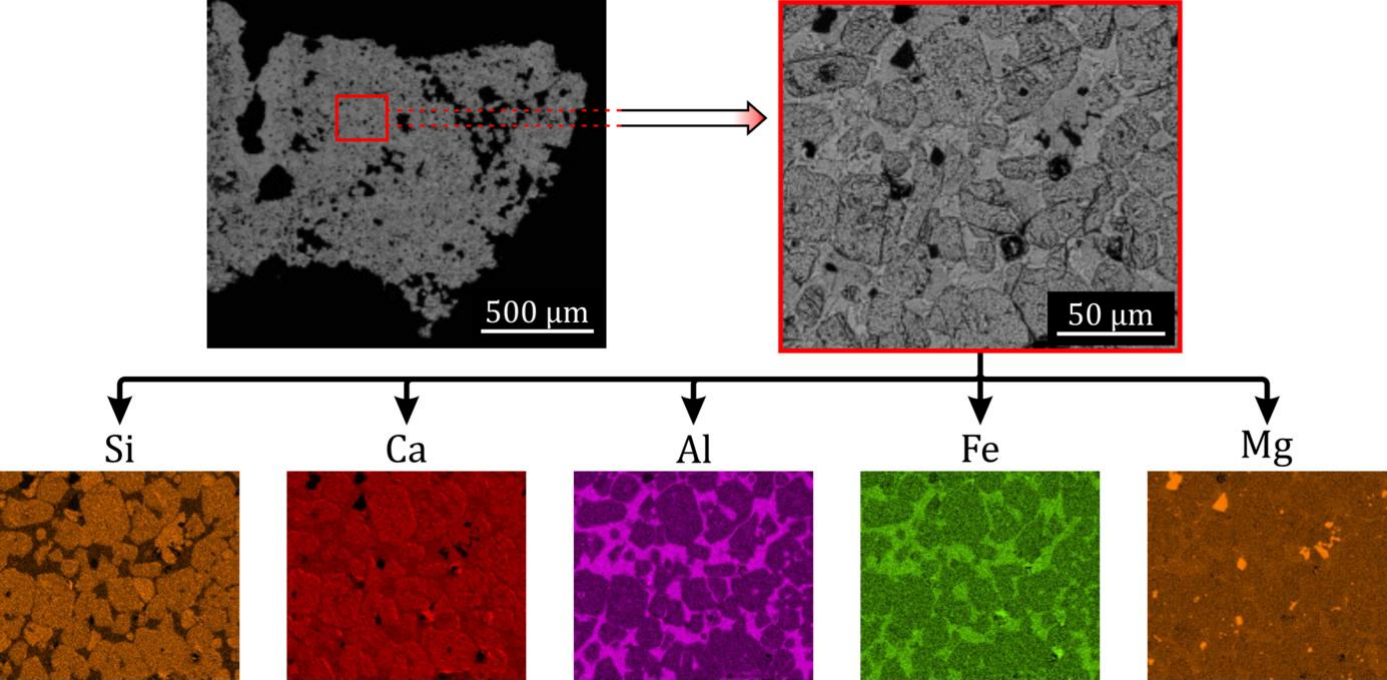
SEM images and chemical mapping of a typical clinker sample. Silicon and calcium energy-dispersive X-ray spectroscopy maps allow the identification of alite (Ca_3_SiO_5_) as the large grains and belite (Ca_2_SiO_4_) as the small ones, since the Si and Ca signals are lower and higher, respectively, for alite.

The main component of cement is clinker, an aggregate of different crystallographic phases. Alite (Ca_3_SiO_5_; 50–70%), belite (Ca_2_SiO_4_; 15–30%), aluminate (Ca_3_Al_2_O_6_; 5–10%) and ferrite (Ca_2_AlFeO_5_; 5–15%) are its main constituents and the different phase ratios determine the strength development of the resulting cement. Several other chemical components like alkali sulfates or calcium oxides are also present in much lower amounts (Taylor, 1997 ▸). Fig. 1 ▸ shows scanning electron microscopy (SEM) images and chemical maps of a typical clinker sample. Its microstructure can be described as large alite and small belite grains crystallized in different polymorphs inside a matrix of ferrites and aluminates, which in turn crystallize in the brownmillerite phase with varying Al/Fe ratios (Kim *et al.*, 1992 ▸; Richardson *et al.*, 1993 ▸; Redhammer *et al.*, 2004 ▸; Dunstetter *et al.*, 2006 ▸).

Alite and belite constituents can crystallize in different phases with closely related cell parameters and space groups. Almost all these crystal structures were determined from X-ray diffraction methods, but single-crystal studies had to be done with crystalline domains grown under laboratory conditions since powder investigations on commercial clinkers or cement samples resulted in diffractograms with severe overlapping of reflections. Although this procedure provides an accurate way of properly characterizing these structures, it may hinder other crystallographic effects produced in the manufacturing process. On the other hand, powder X-ray diffraction (PXRD) gives very precise phase quantifications of the different crystal phases inside the mixture, but it requires the input of all the structures that are thought to be inside to run the Rietveld method (De la Torre & Aranda, 2003 ▸). In this context, transmission electron microscopy (TEM) appears as an alternative and complementary characterization tool that can give very useful insights.

TEM has been used to visualize morphology, twinning and crystalline structures since the initial studies on cement (Remy *et al.*, 1995 ▸). Electron diffraction was frequently used to identify superstructure reflections and suggest different hypothetical structures that were subsequently fitted in PXRD or single-crystal X-ray diffraction (SCXRD) patterns (Jelenić & Bezjak, 1982 ▸; Thompson *et al.*, 1987 ▸; Fukuda & Maki, 1989 ▸). However, electron diffraction analyses were restricted to pure geometric descriptions and its potential for further crystallographic investigations was untapped due to the lack of necessary tools.

In this work, the use of three-dimensional electron diffraction (3D ED) (Kolb *et al.*, 2007 ▸, 2008 ▸; Gemmi *et al.*, 2019 ▸) with an automated data acquisition approach is presented as a new and reliable way of studying the different crystalline phases of clinkers. Such a methodology allows the accurate identification of crystallographic features from individual crystals inside phase mixtures that may be difficult to observe and properly analyse in PXRD, thus increasing the reliability of the available models used later in the quantification of powder patterns. In particular, this work has focused on the investigation of the belite polymorphs present in different commercial clinkers.

## 2. Materials and methods

### 2.1. The different polymorphs of belite

**Figure 2:**
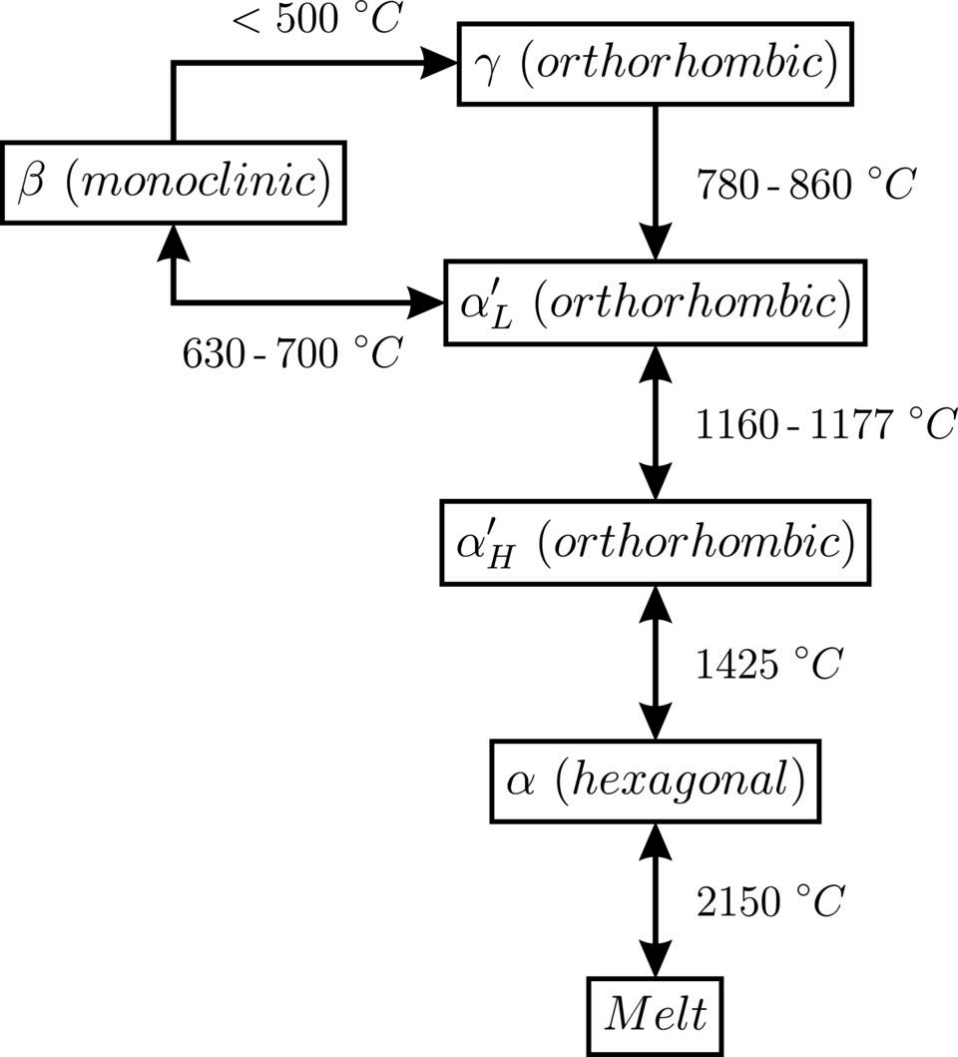
A schematic presentation of the belite (C_2_S) phase transitions between the different polymorphs with respect to temperature (Kim *et al.*, 1992 ▸; Taylor, 1997 ▸).

Belite is one of the major constituents of clinker, thus the structural characterization of its different polymorphs is key to foresee and understand the performance of the resulting cement. Fig. 2 ▸ shows a scheme of the different crystal structures that belite (C_2_S) transitions to according to the temperature.

The only phase that is thermodynamically stable at room temperature is the orthorhombic γ-C_2_S (Fukuda & Maki, 1989 ▸). All other polymorphs require the addition of dopants that partially substitute the silicon by phosphorus and/or the calcium by potassium or strontium, as well as the precipitation of minor oxides that induce strain in the crystal structure (Morsli *et al.*, 2007 ▸). In the case of the clinker manufacturing process, belite grains contain these different crystal phases and the interest is in preserving the high-temperature polymorphs because of their higher reactivity at room temperature. Table 1 ▸ summarizes the crystallographic information on C_2_S polymorphs according to the literature.

The γ polymorph is an orthorhombic crystal that resembles the olivine-type structure and contains two independent calcium cations surrounded by six oxygen environments (Udagawa *et al.*, 1980 ▸). When the temperature is increased, different superstructures are obtained that are generally referred to as 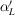 (Regourd *et al.*, 1968 ▸; Il’inets & Bikbau, 1990 ▸). These can be understood as intermediate phases between γ and 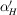 since their superstructure can be derived from the basic unit cell of 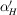 , where oxygen and calcium have split positions and the calcium cations are surrounded by eight or ten oxygen environments (Mumme *et al.*, 1995 ▸). The α polymorph crystallizes in the hexagonal or trigonal crystal system and, in this case, six and seven oxygen-coordinated environments for the calcium cations are observed (Udagawa *et al.*, 1980 ▸). When the temperature is decreased from 700°C or higher to around 600°C, the monoclinic phase is formed with calcium coordinated by six or eight oxygen anions. If the temperature is then decreased further down to room temperature, the γ form is retrieved once again. If enough dopants are added or the belite crystals are sufficiently small, the β polymorph is stabilized and it is prevented from reducing to γ. Since the γ crystal phase is less dense, the clinker production process tries to minimize this transformation because it would produce a more voluminous powder on cooling that results in less strength, an effect called dusting (Taylor, 1997 ▸).

The α, 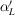 , 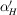 and β phases are classified as glaserite-type structures and they can be subsequently described by the movement of calcium cations and changes in the silicon tetrahedra orientations. The lower the temperature, the lower the symmetry of the related crystalline phase. The nature of the transition between 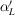 and 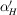 also leads to the observation of modulated features in the form of extra reflections in zone-axis electron diffraction patterns (Saalfeld, 1975 ▸; Jelenić & Bezjak, 1982 ▸; Fukuda & Maki, 1989 ▸). Although these features have been explained using supercells and chemically and crystallographically meaningful descriptions for more than half a century, crystal structure refinements including incommensurate modulations are yet to be explored to comprehend fully the solid-state nature of the compound.

### 2.2. Sample preparation

Three different clinker samples were obtained from the Schwenk Zement KG plant in Bernburg, Germany. They were differentiated by the amount of sewage that was ejected from the burner pipe inside the rotary kiln, namely 0, 2.5 or 5 tonnes per hour, referred in this work as clinker_0, clinker_2.5 and clinker_5, respectively. In all cases, the final amount of sewage used during their production was the same, since the amount that was not shot from the pipe was introduced in the input mixture of the kiln. The ground clinkers were then chemically treated by BASF Construction Solutions GmbH to dissolve the aluminates and ferrites (hydroxide/sugar extraction) in order to easily identify the calcium silicate phases. The resulting powders were dispersed in ethanol and sprayed on TEM carbon-coated copper grids with a UIS250v Hielscher sonifier. The Cu grids were cleaned with argon plasma for one minute before and after the powder was sprayed onto them to eliminate remaining organic compounds and minimize carbon contamination during the TEM measurements.

### 2.3. Data acquisition

The prepared copper grid samples were loaded on an FEI single-tilt holder or a Fischione tomography holder and inserted in an FEI Tecnai F30 field-emission gun operated at 300 kV. The microscope was aligned in scanning transmission electron microscope (STEM) mode with Microprobe illumination. The same data acquisition procedure was carried out for all samples: initial energy-dispersive X-ray spectroscopy (EDS) measurements to identify particles with calcium and silicon, followed by a 3D ED acquisition by means of the *Fast-ADT* routine, an acquisition method developed for the systematic and routine collection of 3D ED data (Plana-Ruiz *et al.*, 2020 ▸). Spot size 6, gun lens 1 and a 50 µm condenser aperture were used to acquire the EDS spectra with an EDAX EDAM III detector. Spot size 6, gun lens 8 and a 10 µm condenser aperture were selected to set a quasi-parallel beam between 150 and 200 nm in diameter to check the particle crystallinity and acquire the diffraction data. STEM images were acquired with a Fischione high-angle annular dark-field (HAADF) detector mounted on the side port of the TEM column and diffraction patterns were collected with a Gatan US4000 with binning 2 and different exposure times. Five crystals were investigated in clinker_0, six in clinker_2.5 and three in clinker_5, from which 3D ED data sets with and without 1° of precession angle were acquired for each particle. Electron beam precession was applied with the DigiStar unit provided by NanoMegas SPRL. Its alignment in STEM mode is described elsewhere (Barnard *et al.*, 2017 ▸; Plana-Ruiz *et al.*, 2018 ▸).

### 2.4. Data processing

EDS data were processed with the *ES Vision* software (Emispec Systems Inc.) to calculate the atom percentage weight of the different crystals. The 3D ED data were reconstructed with the *eADT* program to explore the observable diffraction space, and to determine the unit cells and initial extraction of reflection intensities (Kolb *et al.*, 2019 ▸). *Ab initio* structure determinations were carried out with *SIR2014* from the *hkl* files generated by *eADT* (Cascarano *et al.*, 2010 ▸; Burla *et al.*, 2015 ▸). *PETS2* was used to extract the intensity of reflections from precessed electron diffraction data sets by means of the fitting of a double-peaked curve function (Palatinus *et al.*, 2019 ▸) for further refinements. Kinematical and dynamical refinements were performed with *JANA2006* with these *hkl* files (Petříček *et al.*, 2014 ▸; Palatinus, Petříček & Corrêa, 2015 ▸; Palatinus, Corrêa *et al.*, 2015 ▸). *Ab initio* structure solutions for the modulated structure were obtained from the *SUPERFLIP* program integrated in *JANA2006* (Palatinus & Chapuis, 2007 ▸). Visualizations of the crystal structure models were created using *VESTA 3* (Momma & Izumi, 2011 ▸).

## 3. Results and discussion

### 3.1. Identification of belite polymorphs

EDS spectra allowed a direct identification of the calcium silicate phases, and the quantification of the Ca/Si ratio was accurate enough to distinguish between alite and belite crystal phases (Ca/Si ≃ 3 for alite and Ca/Si ≃ 2 for belite). In this way, 3D ED data sets from suspected belite crystals were analysed for polymorph identification. Table S1 in the supporting information provides the acquisition and output parameters of successful *ab initio* structure solutions by direct methods. Three, one and two β-C_2_S single crystals were identified in clinker_0, clinker_2.5 and clinker_5, respectively, and one 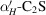 single crystal was found in each sample. The unit cells of these nine crystals were determined from the data sets collected without precession (Table 2 ▸). Unit-cell parameters in 3D ED tend to be inaccurate and a scale correction to their absolute values has been applied. The scale factor was obtained by calculating the average ratio between the unit-cell parameters of the best quality β-C_2_S data set and the values reported by Jost *et al.* (1977 ▸). The β polymorph was used for this scaling as its crystal structure is better understood than the 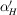 phase. One scale factor was retrieved and applied for each clinker sample.

**Figure 3:**
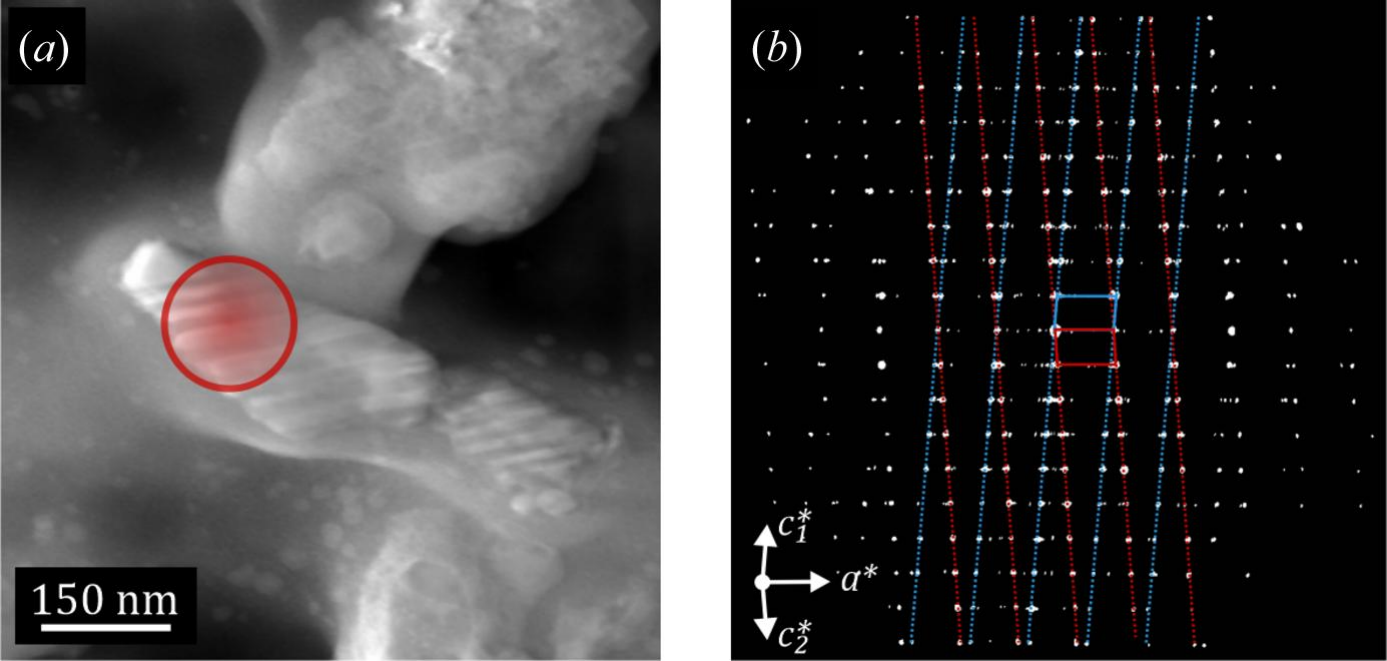
(*a*) A STEM-HAADF image of a twinned β-C_2_S crystal. The red circle marks the region illuminated by the electron beam for the acquisition of the diffraction data set. (*b*) A projection of the reconstructed space along the b* axis acquired from the twinned β-C_2_S crystal. The red and blue overlapping rhomboids represent the projected monoclinic unit cells for the two differently oriented cells, and the dashed lines are displayed for clarity to show which reflections belong to the different twins.

The reconstruction of the observable diffraction space on the β-C_2_S data sets revealed that all measured specimens contained at least one crystal with a typical non-merohedral twin along the [100] direction of the monoclinic cell. Fig. 3 ▸ shows a STEM-HAADF image of one of the measured twinned crystals and the projection along the b* axis of one reconstruction. The diffraction contrast in the STEM image enables the direct visualization of the twin boundaries, and two monoclinic unit cells with the same unit-cell parameters but differently oriented are needed to index all reflections.

Reflection intensity integration and extraction of the β-C_2_S data sets was carried out with *eADT*. In the case of twinned crystals, two separate *hkl* files were obtained for each unit cell. The observation of the reflection condition *h* + *l* = 2*n* in the *h*0*l* reconstructed section pointed to the already reported *P*12_1_/*n*1 space group. Structure solution via direct methods and a kinematical refinement performed with *SIR2014* provided in all cases the crystal structure in the literature (Jost *et al.*, 1977 ▸). Interestingly, since the twinned crystal data sets have low reflection overlap, which is mainly seen at low diffraction data resolutions, both *hkl* files in the three cases result in successful structure determination without significant structural differences between them.

**Figure 4:**
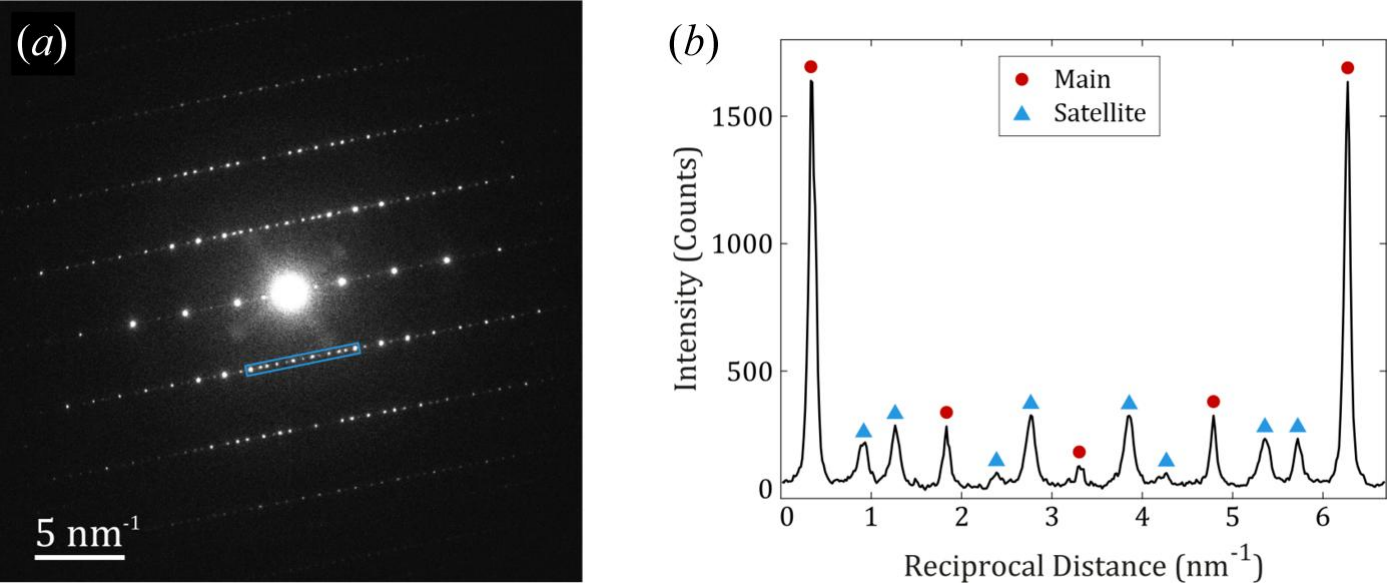
(*a*) A precessed diffraction pattern along [ 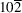 ] from one of the 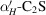 crystals that shows strong extra reflections, and (*b*) an intensity histogram of ( 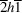 ) that corresponds to the blue region marked in panel (*a*).

In the case of 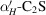 data sets, the reconstructions revealed extra reflections that do not fit the reported 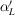 superstructure or 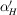 structure. These extra reflections are located along the b* axis of the *Pmnb* setting at ±0.3725, ±0.3795 and ±0.3563 of 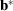 for clinker_0, clinker_2.5 and clinker_5, respectively. The values tend to approach an irrational number, thus large supercells would be required in order to have lattices that fit all observed reflections well, yet the number of structural parameters would be too large and the description would not be accurate. Such features were initially reported by Jelenić & Bezjak (1982 ▸), who identified extra reflections at ∼3/8 and ∼5/8 from the main ones. However, no further structural investigations were undertaken. If these reflections are momentarily ignored, the systematic absences in the *h*0*l* and *hk*0 reconstructed sections confirm the *Pmnb* space group, and the retrieved structure solutions via direct methods correspond to the crystal structure reported by Mumme *et al.* (1995 ▸). Nevertheless, the satellite reflections are not weak, as is shown in Fig. 4 ▸, and they cannot be ignored for a proper crystal structure determination. For this reason, the procedure to determine the crystalline phase must include the significant incommensurate characteristic of the acquired diffraction patterns.

### Crystal structure of belite 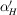


3.2.

Satellite reflections were identified in the diffraction data sets of the 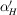 polymorph for each clinker sample. However, the clinker_5 data exhibit textured reflections as well as strong background intensity, decreasing the quality of the reflection intensities. For this reason, this data set has been excluded from the following crystallographic analysis.

The *Pmnb* setting was used in previous sections to enable easy visualization of the phase transitions between the glaserite-type structures of belite. Here the orientation matrix of the standard *Pnma* setting is chosen, and the modulation wavevectors become 0.3725a* and 0.3795a* for the clinker_0 and clinker_2.5 data sets, respectively.

#### 3.2.1. Average structure model

**Figure 5:**
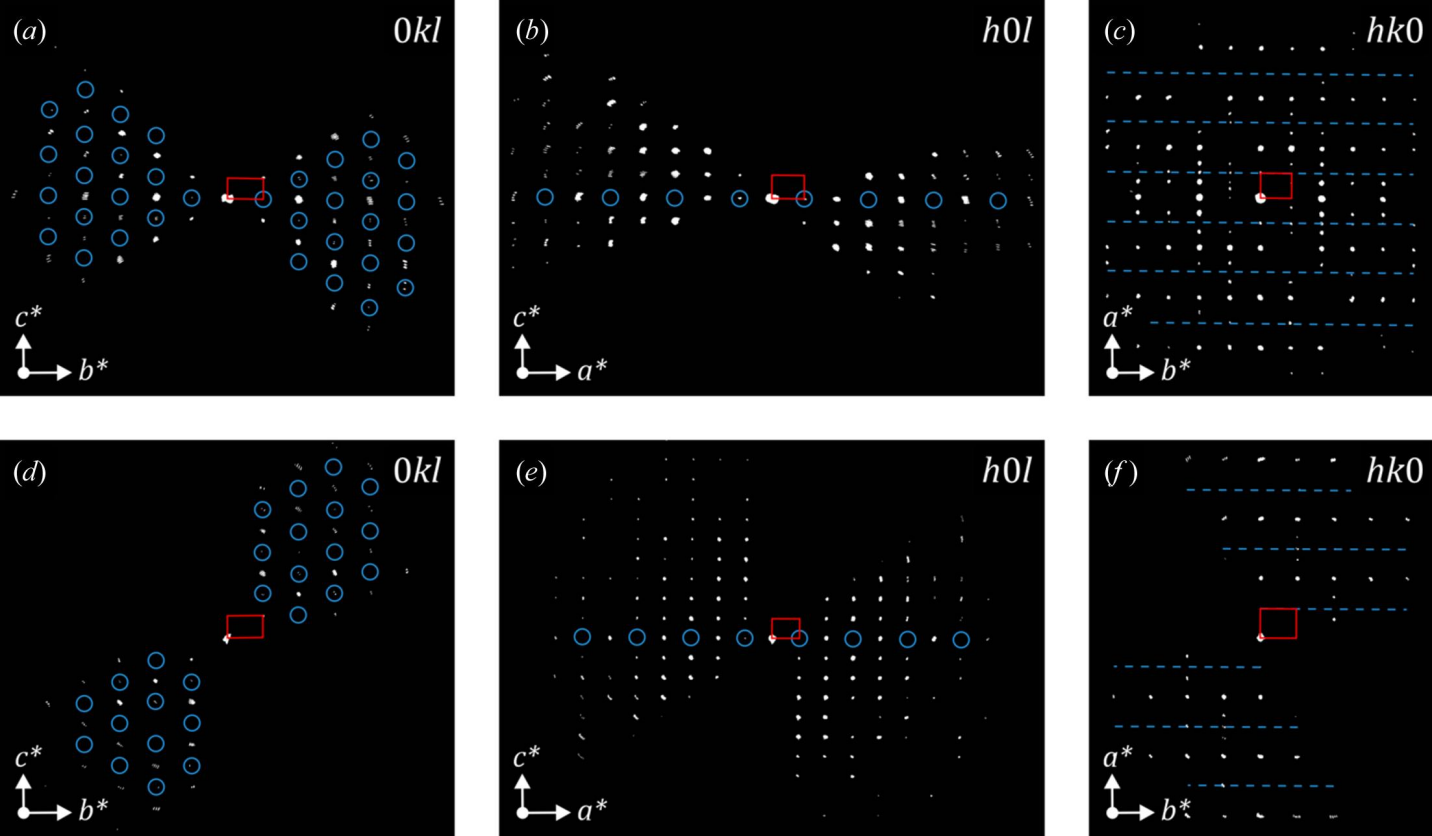
0*kl*, *h*0*l* and *hk*0 sections of the reconstructed space from clinker_0 (upper figures) and clinker_2.5 (lower figures). The red rectangles correspond to the projected unit cells along the different directions of the sections. The blue circles and dashed lines mark the positions of the systematic extinctions according to the *Pn*-*a* extinction symbol.

First, the structure analysis of 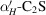 is carried out by ignoring the satellite reflections. The 0*kl*, *h*0*l* and *hk*0 sections displayed in Fig. 5 ▸ show the reflection conditions related to the extinction symbol *Pn*-*a*, *i.e.* the *n*-glide plane perpendicular to the *a* axis that produces the *k* + *l* = 2*n* condition in the 0*kl* section, and the *a*-glide plane perpendicular to the *c* axis related to the *h* = 2*n* condition in the *hk*0 section. The resulting extinction symbol points toward the *Pn*2_1_ *a* and *Pnma* space groups as possible candidates.

The reported crystal structures of 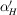 are always in the *Pnma* space group (Regourd *et al.*, 1968 ▸; Saalfeld, 1975 ▸; Mumme *et al.*, 1995 ▸). These models place the calcium and two oxygen positions on the mirror plane or slightly shifted from it and half occupied to compensate for the creation of the symmetrically related atom on the other side of the plane. For this reason, a structure solution with the *Pn*2_1_ *a* space group is a reasonable assumption because it allows these atoms to sit at any specific position along the *b* axis without the limitation of the mirror symmetry. In this way, structure solutions were obtained from direct methods and the retrieved models were refined by three cycles of the least-squares refinement routine in *SIR2014*.

The resulting models from direct methods provided less distorted silicon tetrahedra for the *Pnma* solutions than the *Pn*2_1_ *a* ones. While the silicon to oxygen distances are ranged between 1.54 and 1.61 Å in the *Pnma* structure, the range is increased up to 1.42–1.87 Å for the *Pn*2_1_ *a* case. Kinematical refinements were carried out in *JANA2006* to check whether the distortions observed in the *Pn*2_1_ *a* model are minimized by the least-squares procedure, but the geometrical distortions in the tetrahedra were greatly increased so these structure models were disregarded from further crystal refinement. On the other hand, the structure models with the *Pnma* space group provided good tetrahedral geometry and the Si—O distances ranged between 1.57 and 1.69 Å. The difference Fourier maps in the first refinement executions showed extra potentials around the oxygen positions placed on the mirror plane (4*c* Wyckoff position), so they were moved out of the symmetry-limited position and the occupancy factor was set to 0.5. Extra spherical potential was also observed close to the oxygen atom on the 8*d* Wyckoff site in both data sets that indicated their splitting. In this case, an extra oxygen atom was added and the occupancy factors were set to 0.5 for each atom of the pair. During the refinement, the atomic displacement parameters (ADPs) were restricted to be the same for both oxygen atoms and their total occupancy was restricted to 1. The occupancy and ADP of a single atom was then refined. Table 3 ▸ shows the figures of merit for these kinematical refinements in which the ADPs turned out positive and low, and the least-squares procedure converged.

The structure models obtained from the kinematical refinements were then used for dynamical refinements to refine the anisotropic ADPs and obtain a more accurate description of the crystal. The obtained *R*_1_(obs) values of 0.0557 and 0.0550 for the clinker_0 and clinker_2.5 data sets, respectively, demonstrate the reliability of the retrieved structure model.

**Figure 6:**
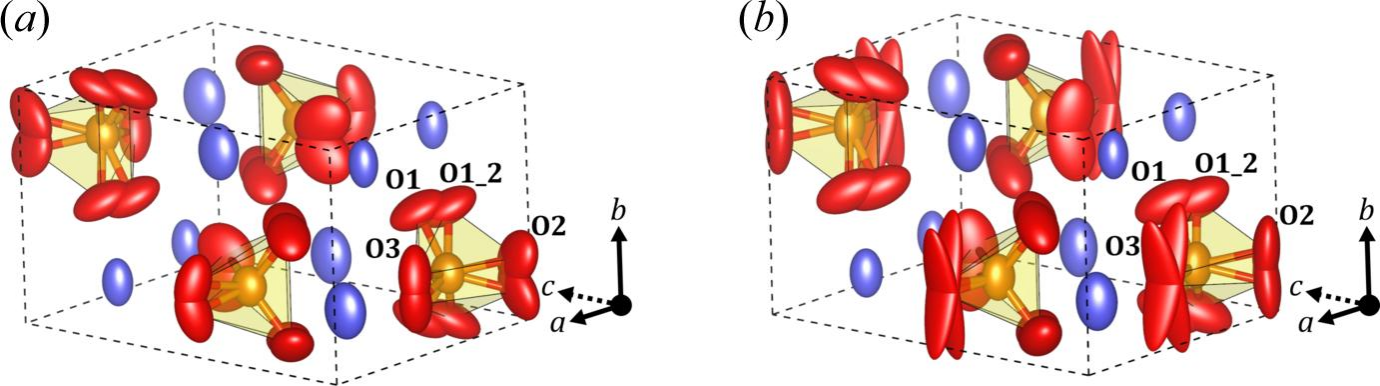
Averaged structure models of 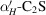 after dynamical refinements from (*a*) the clinker_0 and (*b*) the clinker_2.5 data sets. Purple atoms correspond to calcium, orange ones to silicon and red ones to oxygen. Atom volumes are scaled according to the principal components of the anisotropic ADPs.

The refinement of the 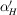 model from the clinker_0 sample results in positive principal components of the anisotropic ADP tensors and the equivalent isotropic ADPs (*B*_iso_) range between 1.2 and 2.4 Å^2^, indicating a reliable positioning of the atoms. The visualization of atom volumes scaled according to the anisotropic ADPs in Fig. 6 ▸ shows that split oxygen atoms near the mirror planes (placed at *y* = 0.25 and *y* = 0.75) have a displacement along the *b* axis, and the remaining pair of split oxygen atoms tend to move to each other while the oxygen to silicon distance is maintained. Calcium atoms also display a movement parallel to the *b* axis, although less severe, and silicon atoms do not exhibit such significant anisotropic displacement. Interestingly, atom O1 has a higher occupation factor than its split pair, O1_2, which points towards a preferred positioning across the cells.

The dynamical refined model taken from clinker_2.5 also results in positive principal ADP components, but atom O3 exhibits a significantly higher component along the *b* axis (Fig. 6 ▸). The small distance between these split oxygen atoms in comparison to the crystal structure of clinker_0 indicates that the ADPs and the pair distance along the *y* component are correlated, yet the least-squares procedure estimates these values to minimize the *R*_1_ value. Furthermore, the data set does not cover either the *b* or *c* axes (Fig. 5 ▸) and, consequently, the resulting electrostatic potential is spread along these directions. Therefore, the high ADP could be explained as an effect of the symmetry-constrained position, missing diffraction space information and the use of an average structure to describe the modulation. In the case of atom O2, the pair distance is higher and the resulting ADP through the *b*-axis direction is not that strong. The left oxygen pair and the calcium positions show the same behaviour as the model from clinker_0, but silicon has a slightly higher displacement along the *b* axis. The higher occupation factor for atom O1 than for O1_2 also suggests the preferred positioning of this oxygen atom seen in the structure from clinker_0.

The very similar refined crystal structures determined from these two different diffraction data sets illustrate that the structure model is reliable, and it can be used to analyse clinker mixtures manufactured under different conditions. Nonetheless, the significant anisotropic behaviour of the found ADPs indicates that a strong structural modulation is present in the crystalline material, which at the same time is observed in the strong satellite reflections. Therefore, the shown models can only be considered as the average structure and the incommensurate characteristic of the diffraction data needs to be taken into account for a proper crystal structure description.

#### 3.2.2. Characterization and refinement of the modulated structure

The *hk*0 sections in Figs. 5 ▸(*c*) and 5 ▸(*f*), as well as the projections of the reconstructed observable diffraction space in Fig. S1, show that the satellite reflections cannot be ignored because of their strong intensity, thus the superspace formalism needs to be used for a correct crystal structure characterization.

The indexing of all reflections in the reconstructed space requires the expansion of the 3D space to a (3+*d*)D superspace. In the presented case, a (3+1)D superspace is required since there is only one modulation wavevector along the a* axis, 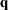 = 0.3725a* for clinker_0 and 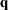 = 0.3795a* for clinker_2.5 ( 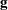 = *h* a* + *k* b* + *l* c* + *m* **q**). First-order satellites are clearly seen in both data sets, but second-order ones do not appear, hence *m* is restricted to 1, leading to an almost 100% indexing of all observed reflections.

The *Pnma* space group identified in the average structure is a good starting point because the main reflections correctly follow its reflection conditions. The *International Tables for Crystallography* Vol. C (Prince, 2004 ▸) show two possible superspace groups for the space group number 62: 62.1, *Pnma*(00γ)000, and 62.2, *Pnma*(00γ)0*s*0. In the first one, the superspace symmetry operations do not have any glide components along the additional dimension, while the second one applies a 1/2 translation along the fourth superspace coordinate for the mirror plane perpendicular to 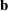 . Although not listed, *Pnma*(00γ)*s*00 and *Pnma*(00 γ)*ss*0 are also possible and they have to be considered. If these superspace groups are taken into account with the modulation vector along the a* axis, the subsequent reflection conditions according to the different superspace groups are identified and listed in Table 4 ▸. Such conditions can now be evaluated in the reconstructed sections shown in Fig. 5 ▸ but using the four indices, *hklm*.

**Figure 7:**
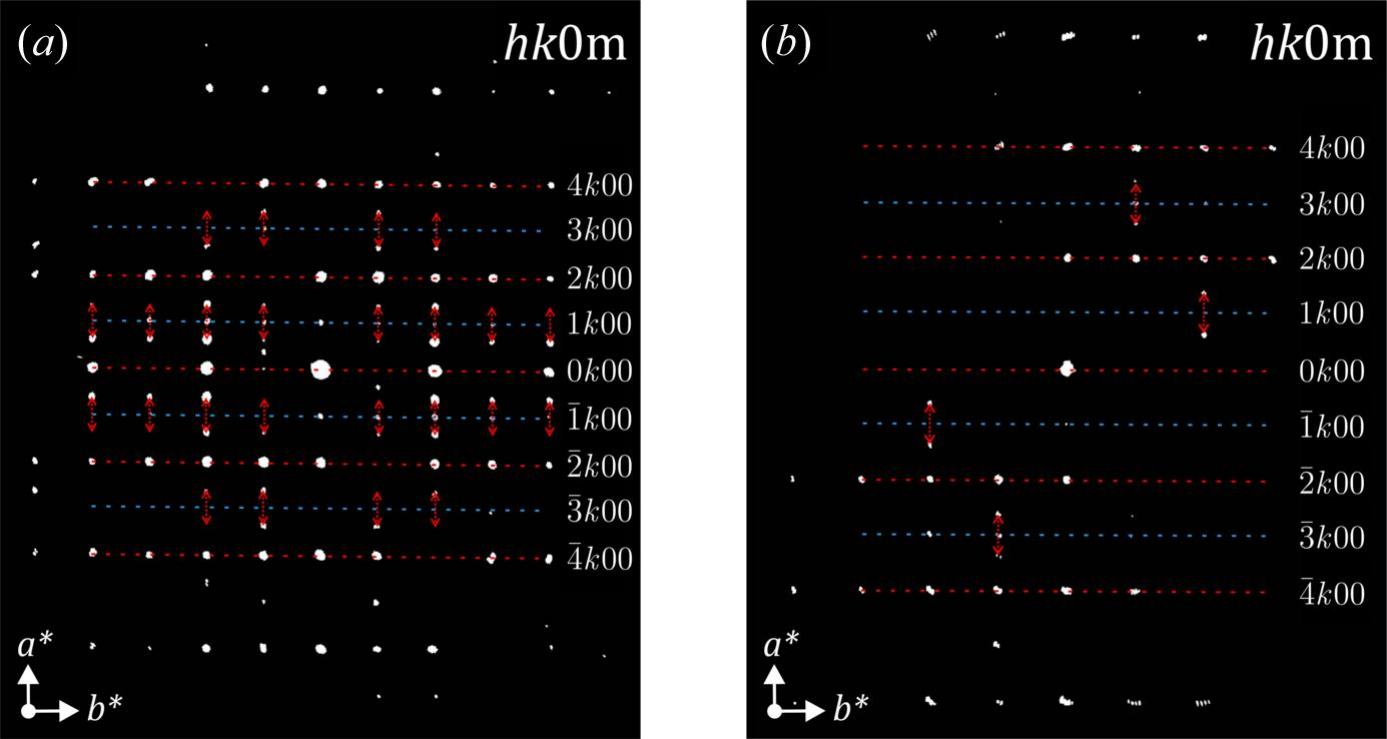
*hk*0*m* sections from the reconstructions of the observable diffraction space that correspond to (*a*) the clinker_0 and (*b*) the clinker_2.5 diffraction data sets. Red and blue dashed lines correspond to *hk*00 rows with even and odd *h* indices, respectively. Red dashed arrows point to the visible satellite reflections from their closest main reflection.

**Figure 8:**
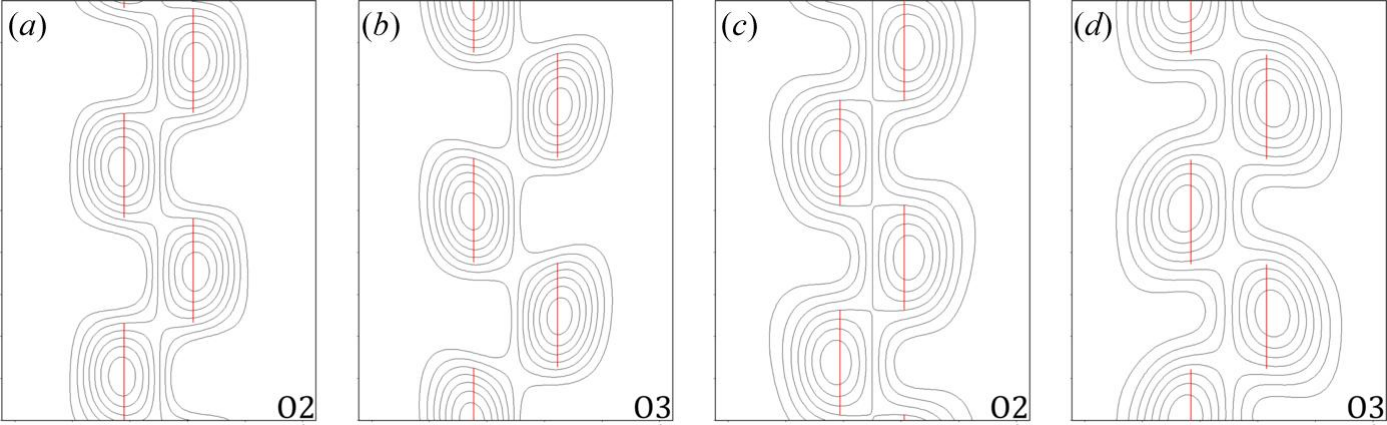
(*x*_*s*,2_, *x*_*s*,4_) de Wolff sections of O2 and O3 domains from (*a*)–(*b*) clinker_0 and (*c*)–(*d*) clinker_2.5. The vertical axis corresponds to *x*_*s*,4_ (*t* = [0, 2]) and the horizontal axis to *x*_*s*,2_. Red lines correspond to the dynamical refined crenel functions assigned to the atomic domains.

The *h*0*lm* sections of Figs. 5 ▸(*b*) and 5 ▸(*d*) clearly show that no satellite reflections are visible, therefore the *h*0*lm*:*m* = 2*n* condition is fulfilled because the fourth index *m* is limited to ±1 and should be visible in this plane. The *hk*0*m* sections displayed in Fig. 7 ▸ show that the *h* = 2*n* condition for the main reflections of the sub-plane *hk*0 is fulfilled, *i.e.* there are no significant strong reflections along the blue dashed lines. This indicates the presence of the *a*-glide plane perpendicular to the *c* axis. However, satellite reflections are observed here and almost all of them appear around the reflections in the *hk*00 rows with *h* = 2*n* + 1 (marked by red dashed arrows in Fig. 8 ▸). Some weak reflections violate these conditions, but this is most probably a result of dynamical effects. When considering these two reflection conditions, the *h* + *m* = 2*n* condition is obtained for the *hk*0*m* plane. From the symmetry point of view, a 1/2 translation along the fourth superspace coordinate for the *a*-glide plane perpendicular to 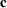 has to be added to the 1/2 translation for the mirror plane perpendicular to 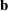 identified in the *h*0*lm* plane. Therefore, the superspace group for the incommensurately modulated structure of 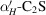 should be *Pnma*(00)0*ss*.

At this point, main and satellite reflection intensities were integrated and extracted from *PETS2*, and the obtained *hklm* reflection files were imported to *JANA2006*. First, the charge-flipping algorithm implemented in *SUPERFLIP* was used to obtain structure models that directly contained a first-order harmonic function for each atomic domain, as this algorithm is able to retrieve electrostatic potentials in an *n*-dimensional space (Palatinus & Chapuis, 2007 ▸; Palatinus, 2013 ▸). One of the oxygen atoms on the 4*c* position was missing in both diffraction data sets but they were found by a Fourier synthesis. It is worth noting that the Fourier synthesis also considers the 4D space, thus the suggested domains and their related averaged positions include modulation parameters as well. Both initial structure models were retrieved with two of the oxygen atoms placed on 4*c* positions. After all atomic positions were found, a first kinematical refinement was done with unconstrained modulation parameters but fixed isotropic ADPs. Inspection of the (*x*_*s*,1_, *x*_*s*,4_), (*x*_*s*,2_, *x*_*s*,4_) and (*x*_*s*,3_, *x*_*s*,4_) de Wolff sections (de Wolff, 1974 ▸) for all atoms revealed that a harmonic function fits well for calcium, silicon and the oxygen atom on the 8*d* site, yet the oxygen atoms on the mirror plane exhibit discontinuous trends in the (*x*_*s*,2_, *x*_*s*,4_) de Wolff section. This suggests that the displacive modulation of O2 and O3 atomic domains could be better described by crenel functions. In this way, the *x*_*s*,2_ and *x*_*s*,4_ coordinates of the maximum peaks in the (*x*_*s*,2_, *x*_*s*,4_) de Wolff sections for O2 and O3 were used to place these atomic domains out of the mirror plane (shift along the *b* axis and occupancy set to 1) and assign crenel functions with 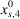 parameters initially set to the found *x*_*s*,4_ coordinates for subsequent refinements and Δ = 0.5. The symmetry of the superspace group ensures that there is no discontinuity along the direction of *x*_*s*,4_ for these atomic domains while they follow discontinuous functions. Fig. 8 ▸ shows the (*x*_*s*,2_, *x*_*s*,4_) de Wolff sections of O2 and O3 for both diffraction data sets and Fig. S2 shows all other de Wolff plots.

Once the modulation functions were clear, further least-squares refinements were performed. First, the modulated model was refined with free modulation parameters (except Δ) and fixed isotropic ADPs. After convergence, the modulation parameters were fixed and the isotropic ADPs were refined, which led to convergence and small and positive ADPs. Finally, all structural parameters were refined and the least-squares procedure converged with well fitted functions to the de Wolff sections and physically meaningful isotropic ADPs. These kinematical refined models were then used as input to dynamical refinements (Palatinus, 2017 ▸). The same refinement workflow as the kinematical case was followed, but the optimization of the orientation angles from all patterns according to the calculated dynamical reflection intensities was carried out prior to the last refinement execution with all structural parameters free. Table 5 ▸ shows the resulting figures of merit from the dynamical refinements of the two data sets, and Tables S2 and S3 show the structural parameters for both refined incommensurately modulated structures. Low isotropic ADPs, well fitted modulation functions and good *R*_1_ values considering main, satellite or all reflections demonstrate the reliability of the determined crystal structure.

#### 3.2.3. Crystal structure description

The reference crystal structure of 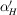 from Mumme *et al.* (1995 ▸) shows that all atoms except silicon are split into half-occupied positions. However, no satellite reflections were reported in that publication, most likely because of the high population of reflections in the PXRD. The electron diffraction structure analysis carried out here demonstrates that the atomic position splitting observed in powder diffraction comes from the intrinsic incommensurate modulation of the crystal structure. In fact, the average structure retrieved using only the main reflections coincides with the X-ray model without the splitting of the calcium positions. It is worth noting as well that the obtained unit-cell parameters are very similar between the two data sets of electron diffraction, but also to the X-ray structure, which indicates that this crystal structure can be reliably used for phase quantifications in powder patterns of different cement samples [data from Mumme *et al.* (1995 ▸): *a* = 6.7673 Å, *b* = 5.5191 Å and *c* = 9.3031 Å; this work, clinker_0: *a* = 6.776 Å, *b* = 5.496 Å and *c* = 9.252 Å; this work, clinker_2.5: *a* = 6.765 Å, *b* = 5.514 Å and *c* = 9.250 Å].

Nevertheless, the strong intensities of the satellite reflections observed in both diffraction data sets, as well as the high anisotropy of the ADPs for the average structure, imply that the modulation cannot be ignored for a proper crystal structure determination. Inspection of the reconstructed observable diffraction space pointed to the superspace group *Pnma*(α00)0*ss* as the symmetry group that fits to the reflection conditions. The subsequent structure solutions and least-squares refinements in *JANA2006* showed that such a superspace group allows us to solve and refine the crystal structure using harmonic and crenel functions to describe the incommensurate modulation. The high ADPs obtained in the averaged models were not retrieved in the refined modulated structures.

**Figure 9:**
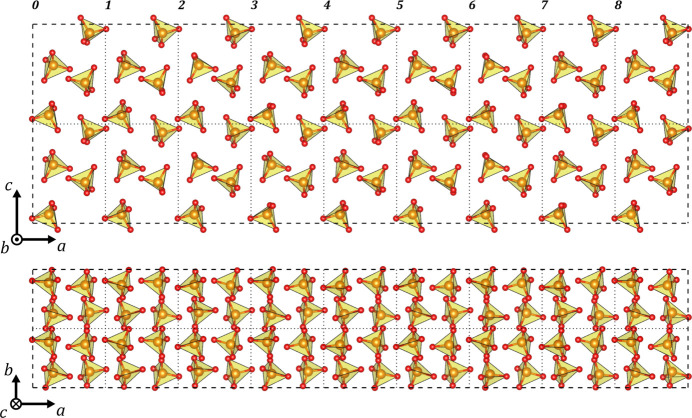
The approximated superstructure with 9 × 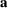 , 2 × 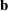 and 2 × 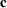 with respect to the unit cell of the incommensurately modulated structure of clinker_0. The upper figure is the projection along the *b* axis, and the lower one is along 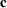 . Calcium atoms have been omitted for clarity of the modulated distortion and orientation of Si–O tetrahedra. Dotted lines are displayed to show the positions of the subcells.

The visualization and description of aperiodic crystals is not trivial because such crystals are represented in a (3+*d*)D space, yet they need to be illustrated back to the physical 3D space (Wagner & Schönleber, 2009 ▸). One way is to build a superstructure with the unit cell of the aperiodic crystal as the subcell of the supercell. Fig. 9 ▸ shows such a superstructure along 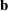 and 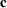 for the clinker_0 refined model (9 × 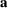 , 2 × 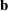 , 2 × 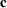 ). Since the modulation wavevector is along the a* axis, the crystal structure preserves the translational symmetry in the *xy* plane, which means that the orientation and geometry of the Si–O tetrahedra as well as the calcium positions can change while going through the *x* component of the unit-cell framework, but the atomic positions translated through the *y* and *z* components are preserved. This can be seen in Fig. 9 ▸, as the tetrahedron positions and geometries in the subcell limited by the dotted lines are the same when shifted to the subcells along 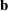 or 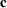 , yet they change along 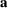 . Another interesting characteristic of the approximated superstructure is that the best supercell to fit all reflections can be identified by checking which subcell most closely resembles the initial one. This coincides with the cell that corresponds to an integer period of the modulation wavevector. In the case of 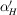 , this is fulfilled after eight subcells and it is shown in Fig. 9 ▸, where the ninth subcell (labelled 8) is the most comparable to the first one (labelled 0).

**Figure 10:**
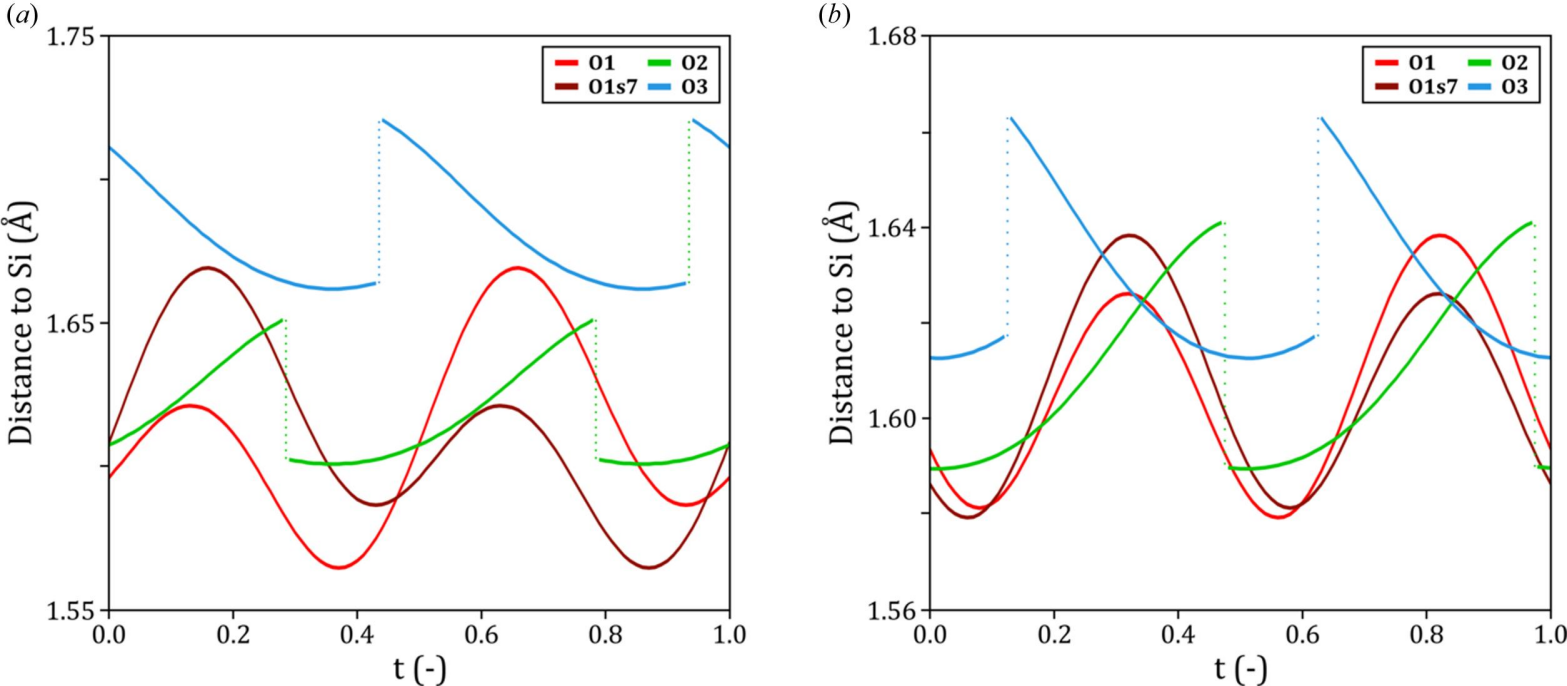
Distances between silicon and the four tetrahedral oxygen atoms with respect to the modulation phase *t*. Panel (*a*) corresponds to clinker_0 and panel (*b*) to clinker_2.5.

The projections of the superstructure in Fig. 9 ▸ graphically show that the geometry of the silicon–oxygen tetrahedra and their orientations change through the *a* axis as a consequence of the modulation of their atoms. A better way of quantifying these modulation characteristics is to plot the interatomic distances with respect to the phase *t* of the modulation wavevector. Fig. 10 ▸ shows the silicon to oxygen distance plots for the models from clinker_0 and clinker_2.5. Atom O3 presents the longer distance to Si, up to 1.721 (6) Å and 1.663 (3) Å for clinker_0 and clinker_2.5, respectively. The harmonic modulation of silicon and the discontinuous positioning of atoms O2 and O3 result in a decrease in the Si—O3 distance and an increase in the Si—O2 distance for every half period of the modulation function, which tend to average distances of 1.661 (4) Å (clinker_0) and 1.627 (3) Å (clinker_2.5) until the oxygen atoms jump to the mirror-related positions. Atom O1 and its symmetry-related oxygen atom O1s7 that completes the tetrahedron have silicon–oxygen curves that are almost in phase between them. This indicates that the harmonic modulation of silicon is in phase to one of these oxygen atoms, while the other is out of phase due to the symmetry relation. Such a particular displacement points towards a silicon to oxygen distance that changes due to the higher displacement value of the oxygen with respect to the silicon atom, but the direction of the silicon–oxygen movement is almost the same for one of the oxygen atoms and the other way around for the symmetry-related one.

The calcium coordination is complicated to illustrate through superstructure projections and interatomic distance plots because of the high number of related oxygen atoms. Nevertheless, careful inspection of both refined structures shows that calcium has octahedral and dodecahedral coordination when Ca—O distances between 2.2 and 3.2 Å are considered. The geometry of these coordination environments changes through the *a* axis and that is why the Ca—O distance for some oxygen atoms is increased to ∼3.2 Å, compared with the ∼2.8 Å distance of the averaged structure.

The use of crenel functions for atoms O2 and O3 confirms that the split and half-occupied atoms in the average structure are a good approximation of the real nature of the crystal structure. However, the rest of the atoms require harmonic functions to describe its intrinsic modulation. Table 6 ▸ reports the distances between split positions for the structure reported by Mumme *et al.* (1995 ▸), the average structures and the total modulated displacements for the modulated structures. These values show that in general the total movement of the atoms in the modulated crystal structure is larger than the reported one on the average structures, especially for atom O1. Silicon also has a displacive modulation that is not directly detected in the average approximations, although much weaker, but which provides a better description of the crystal structure. Finally, it is worth noting that the values of the model from clinker_0 are higher than clinker_2.5, which demonstrates that the modulation is stronger in the first data set, as visible also in the stronger intensities of satellite reflections.

#### 3.2.4. Phase transitions of the belite polymorphs

The structures of the different belite polymorphs can be derived from the hexagonal closest packing of atoms (Bragg & Claringbull, 1965 ▸). The γ phase has an olivine-type structure, the α and β polymorphs are β-K_2_SO_4_-type structures, and 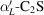 is a superstructure of such a β-K_2_SO_4_-type structure (Udagawa *et al.*, 1980 ▸). When the temperature is increased, CaO_*n*_ polyhedra in the β-K_2_SO_4_-type structures increase in size, while the size of the SiO_4_ tetrahedra does not change (Smyth & Hazen, 1973 ▸). This large size difference leads to tilting, shifting and distortion of the SiO_4_ tetrahedra (Toraya & Yamazaki, 2002 ▸). On the other hand, this size difference is not present in the olivine-type structure of the γ polymorph, which explains why calcium is octahedrally coordinated in γ but has more adjacent oxygen atoms in the other polymorphs, ranging up to a tenfold coordination. A comparable behaviour of metastable phases containing different Ca coordination numbers is vaterite, which was also solved by 3D ED (Mugnaioli *et al.*, 2012 ▸).

**Figure 11:**
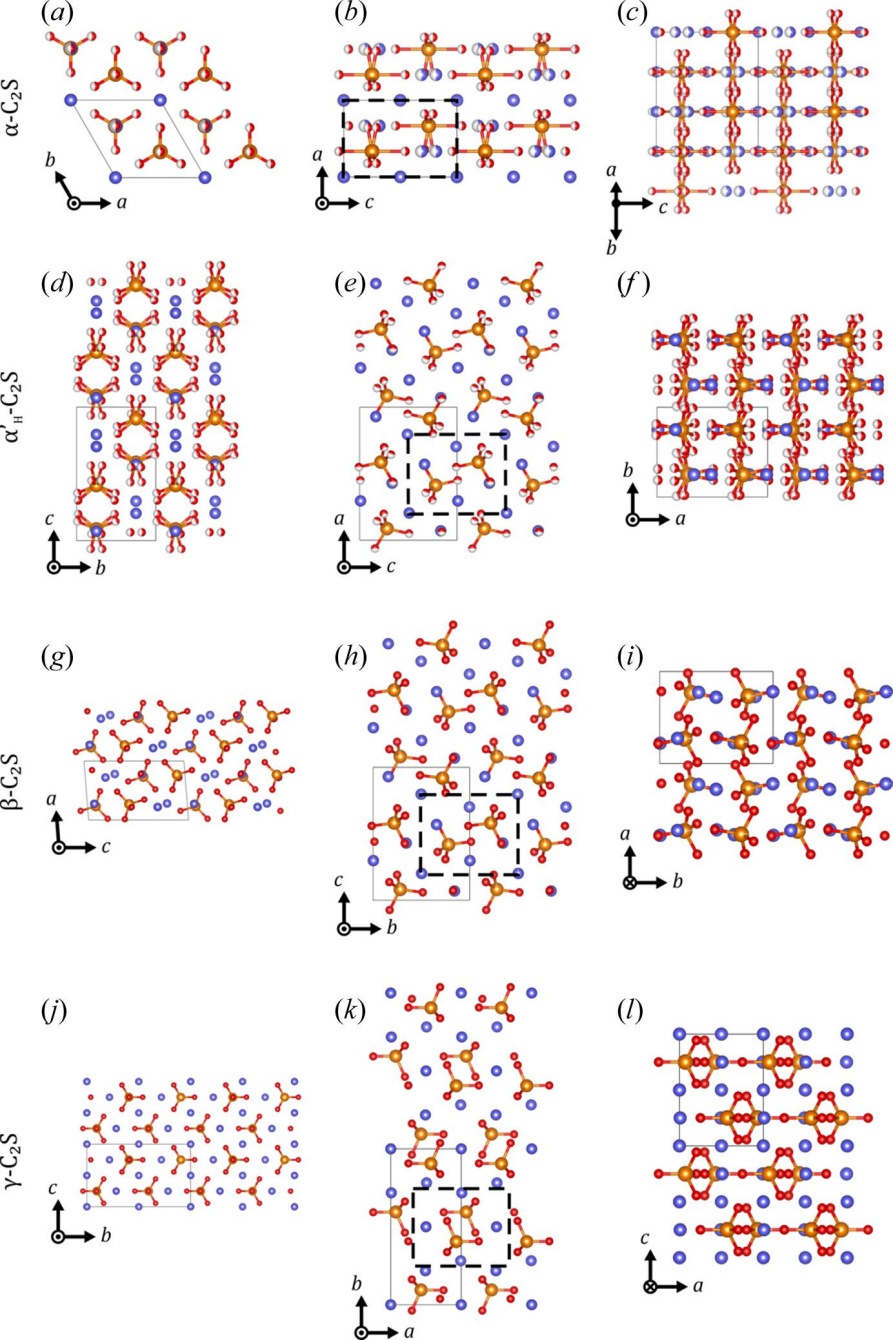
(*a*)–(*c*) The α-C_2_S structure along (*a*) the *c* axis, (*b*) the *b* axis and (*c*) the ( 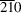 ) direction. (*d*)–(*f*) The average structure of 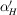 belite along (*d*) the *a* axis, (*e*) the *b* axis and (*f*) the *c* axis. (*g*)–(*i*) The β-C_2_S structure along (*g*) the *b* axis, (*h*) the *a* axis and (*i*) the *c* axis. (*j*)–(*l*) The structure of γ-C_2_S along (*j*) the *a* axis, (*k*) the *c* axis and (*l*) the *b* axis. Panels (*a*), (*d*) and (*g*) correspond to the ∼7 Å direction, (*b*), (*e*) and (*h*) to the ∼5 Å direction, and (*c*), (*f*) and (*i*) to the ∼9 Å direction. The viewing directions of γ-C_2_S are selected accordingly for proper comparison. The projections in (*b*), (*e*), (*h*) and (*k*) display the original cell of α-C_2_S as dashed black lines to illustrate the formation of tetrahedron pairs in 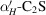 . The structures are displayed as 2 × 2 × 2 of the respective unit cells. Ca is displayed in purple, Si in orange and O in red.

In order to study and illustrate the phase transitions of the different belite phases, three characteristic viewing directions were selected that correspond to the main directions in most belite polymorphs: the ∼5, ∼7 and ∼9 Å axes. Starting with the high-temperature α-C_2_S, the hexagonal axis can be seen along the *c* axis (∼7 Å axis) in Fig. 11 ▸(*a*). In this case, the hexagon is constructed from two mutually displaced triangles of SiO_4_ tetrahedra that are in two different planes along *c*. Along the *b* axis [∼5 Å axis; Fig. 11 ▸(*b*)], the SiO_4_ tetrahedra are arranged in slightly shifted rows through the *c* direction. Along the ( 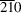 ) direction [∼9 Å axis; Fig. 11 ▸(*c*)], they are arranged in rows running along *b*. Because of the *P*6_3_/*mmc* space group, the oxygen atoms of the SiO_4_ tetrahedra and some Ca positions are split in the direction of the *c* axis.

Upon cooling, the statistically ordered α-C_2_S transitions to the 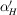 polymorph at 1425°C. Here, the orientation of the cell changes with the transformation matrix [0 0 1; 0 1 0; −2 −1 0] and an origin shift of (0, ¼, ¼). Along the ∼7 Å direction, which originally indicated sixfold symmetry, a slight tilting and twisting of the split SiO_4_ tetrahedra is visible [Fig. 11 ▸(*d*)]. The two triangles that form the hexagon are also slightly tilted with respect to each other. Along the *c* direction (∼9 Å axis), rows of split SiO_4_ tetrahedra can be seen, but every other tetrahedron is rotated with respect to the *b* axis [Fig. 11 ▸(*f*)]. This change in the structure is achieved by a combination of a rotation around 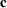 and a slight rotation around 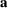 , and an additional ¼ shift in 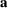 and 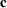 . Along the *b* axis (∼5 Å axis), the splitting of the tetrahedra cannot be seen. The rotation of the tetrahedra results in the formation of tetrahedron pairs in this direction [black dashed rectangle in Fig. 11 ▸(*e*)].

**Figure 12:**
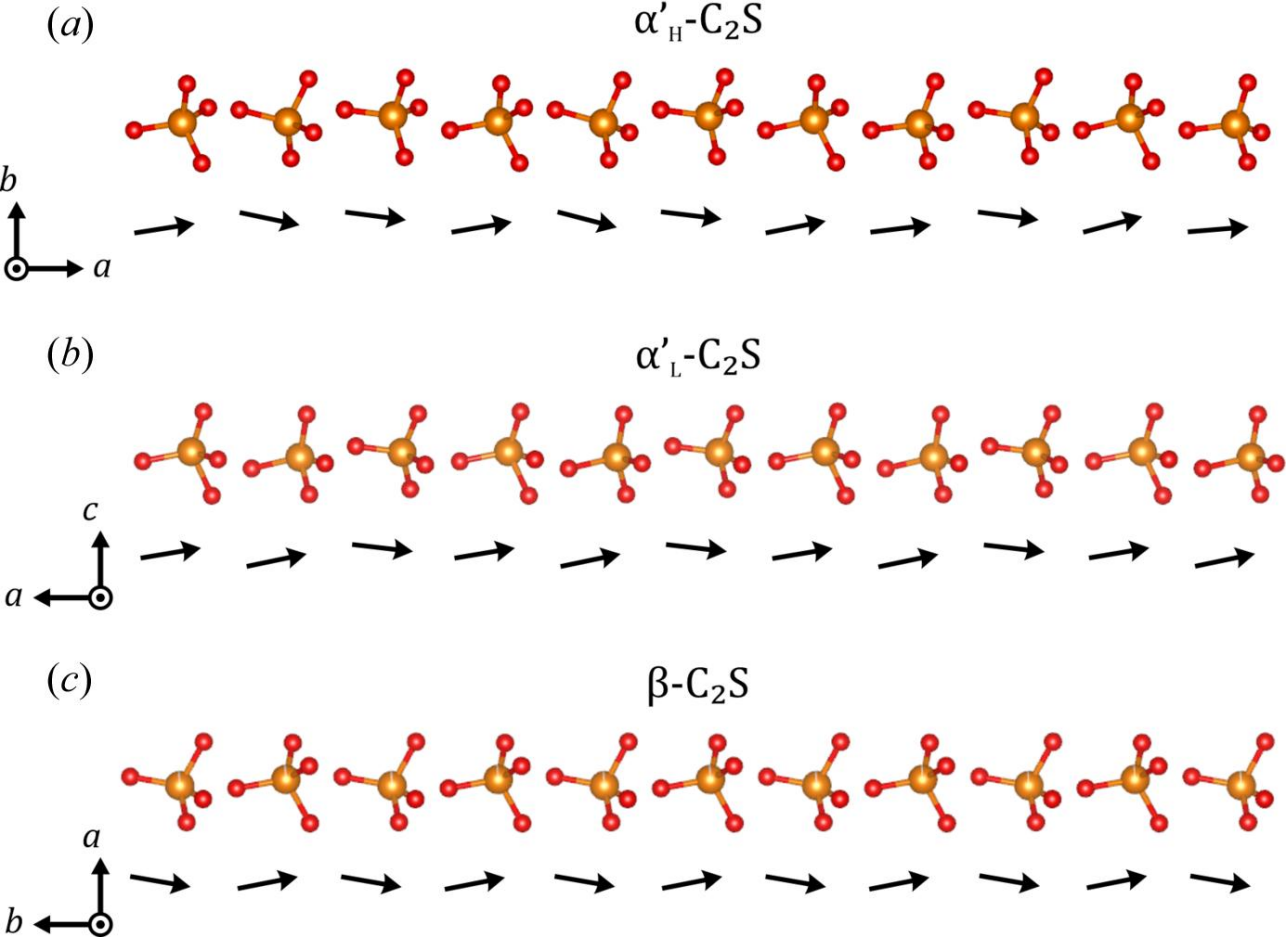
Rows of Si tetrahedra projected along the ∼9 Å viewing direction of (*a*) the incommensurate modulated structure of 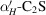 , (*b*) 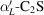 and (*c*) β-C_2_S. The tilt of each tetrahedron is illustrated by the black arrow below it. Ca positions have been omitted for clarity. Si is displayed in orange and O in red.

Upon further cooling, a transformation to 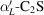 occurs at about 1160–1177°C. In this case, the *b* and *c* axes are interchanged, while the orientation of the *a* axis remains the same. The ordering leads to a change in space group to *Pna*2_1_. The biggest difference with respect to the 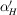 structure can be seen in the ∼9 Å direction. In the incommensurate modulated structure of 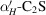 , the Si tetrahedra are tilted irregularly along the *a* axis [Fig. 12 ▸(*a*)]. In the 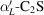 structure the tetrahedra are now ordered along the *a* axis in an up–up–down pattern [Fig. 12 ▸(*b*)], causing a threefold superstructure and explaining the tripling of the 7 Å axis (a* direction), which coincides with the direction of the modulation vector of 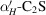 .

Upon further cooling, the structure is transformed to β-C_2_S. In this process, the orthorhombic crystal system is converted into a monoclinic one and the orientations of the axes change according to the transformation matrix [0 1 0; 0 0 1; 1 0 0]. The biggest distortion is seen in the ∼7 Å viewing direction where the original sixfold symmetry is strongly distorted and the distances between the triangles change [Fig. 11 ▸(*g*)]. In the ∼5 Å direction, the tetrahedron pairs remain, but are slightly distorted with respect to each other [Fig. 11 ▸(*h*)]. The rows of tetrahedra observed in the ∼9 Å direction break up [Fig. 11 ▸(*i*)]. In comparison to the 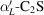 structure, the tilting of the Si tetrahedra changes from an up–up–down to an alternating down–up pattern [Fig. 12 ▸(*c*)].

Transformation to the γ phase, which is stable at room temperature, changes the crystal system back to ortho­rhombic. In this case, however, the structure resembles the olivine-type structure instead of the β-K_2_SO_4_-type structure. These significant changes are caused by large rotational movements around all three axes. Accordingly, the axis lengths also change and no longer correspond to the ones defined above. Instead, the ∼7 Å axis now shows the tetrahedron pairs together [Fig. 11 ▸(*k*)]. Additionally, the tetrahedral hexagon along the ∼5 Å direction is no longer recognizable [Fig. 11 ▸(*j*)]. The greatest change can be seen along the ∼11 Å axis, where the rows of tetrahedra are no longer present [Fig. 11 ▸(*l*)].

## 4. Conclusions

For more than half a century the crystalline characterization of cement samples has been hampered by the difficulties of acquiring diffraction data sets from individual crystals of actual cement powders. The development of the 3D ED technique and systematic acquisition methods in a trans­mission electron microscope, like the *Fast-ADT* routine, have proved in this work to be suitable for the identification and crystallographic analysis of individual particles in clinker powders.

The processing of the acquired diffraction data sets has shown that most acquisitions belong to β-C_2_S, some of which contain the typical non-merohedral [100] twinning of monoclinic crystals. The reconstruction of the observable diffraction space from the 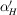 polymorph data sets exhibits an incommensurately modulated component along the *a* axis that was previously reported but never properly characterized. The inspection of the reconstructed space indicates that the structure could be described in the *Pnma*(α00)0*ss* superspace group and *hklm* files were extracted for structure determinations based on the superspace formalism. The subsequent use of harmonic and crenel functions to describe the modulation of all atoms in dynamical refinements results in good figures of merit as well as good fitting of the functions in the de Wolff sections, which confirms the proper crystal structure determination of the novel incommensurately modulated structure.

The fact that two different diffraction data sets from two different manufactured samples result in such close refined structure models indicates that the crystalline description of 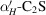 in this work is generally valid for different processed cements. The structure published by Mumme *et al.* (1995 ▸) and the refined average structures reported here show that these models do not deviate strongly from the real crystalline nature of the material, and they can be used as good approximations. Nevertheless, the incommensurately modulated structure provides a better model for this crystalline phase, which can be used in the phase quantifications frequently carried out in PXRD patterns to enhance the reliability of fitting by the Rietveld method.

## Supplementary Material

Crystal structure: contains datablock(s) global, 0k_average_dyn, 0k_modulated_dyn, 2p5K_average_dyn, 2p5K_modulated_dyn. DOI: 10.1107/S205252062400146X/je5054sup1.cif

Structure factors: contains datablock(s) 0K_modulated_dyn. DOI: 10.1107/S205252062400146X/je50540k_modulated_dynsup2.hkl

Structure factors: contains datablock(s) 2p5K_modulated_dyn. DOI: 10.1107/S205252062400146X/je50542p5K_modulated_dynsup3.hkl

Additional tables and figures. DOI: 10.1107/S205252062400146X/je5054sup4.pdf

7bhGderGvHs

CCDC references: 2332749, 2338192, 2338193, 2338194

## Figures and Tables

**Table 1:** Crystal structure information for the different polymorphs of C_2_S obtained by SCXRD or PXRD The adjusted setting is provided for further clarity of the crystal structure description of the polymorph phase transitions (Section 3.2.4).

		Standard setting		Adjusted setting	
Polymorph	Crystal system	Lattice parameters	Space group	Lattice parameters	Space group
γ	Orthorhombic	*a* = 11.214 Å	*Pnma* ^(*a*)^	*a* = 5.081 Å	*Pcmn* ^(*b*), (*c*)^
		*b* = 6.758 Å		*b* = 6.778 Å	
		*c* = 5.076 Å^(*a*)^		*c* = 11.244 Å^(*b*)^	
β	Monoclinic	*a* = 5.502 Å	*P*12_1_/*n*1^(*d*)^	*a* = 5.502 Å	*P*12_1_/*n*1^(*d*), (*e*)^
		*b* = 6.745 Å		*b* = 6.745 Å	
		*c* = 9.297 Å		*c* = 9.297 Å	
		β = 94.59°^(*d*)^		β = 94.59°^(*d*)^	
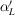 (2*a*, *b*, 2*c*)†	Orthorhombic	*a* = 11.184 Å	*Pbcn* ^(*f*)^	*a* = 11.184 Å	*Pcnb* ^(*f*)^ *Pmnb* or *Pmnn* ^(*g*)^
		*b* = 18.952 Å		*b* = 6.837 Å	
		*c* = 6.837 Å^(*f*)^		*c* = 18.952 Å^(*f*)^	
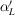 (*a*, 3*b*, *c*)†	Orthorhombic	*a* = 9.500 Å	*Pna*2_1_^(*h*)^	*a* = 5.601 Å	*P*2_1_ *nb* ^(*h*)^ *Pmnb* ^(*i*)^
		*b* = 5.601 Å		*b* = 20.863 Å	
		*c* = 20.863 Å^(*h*)^		*c* = 9.500 Å^(*h*)^	
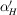	Orthorhombic	*a* = 6.767 Å	*Pnma* ^(*a*)^	*a* = 5.519 Å	*Pmnb* ^(*a*), (*f*), (*i*)^
		*b* = 5.519 Å		*b* = 6.767 Å	
		*c* = 9.303 Å^(*a*)^		*c* = 9.303 Å^(*a*)^	
α	Hexagonal / triclinic	*a* = 5.579 Å	*P*6_3_/*mmc* ^(*b*)^	*a* = 5.579 Å	*P*6_3_/*mmc* ^(*b*)^ 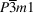 ^(*j*)^
		*c* = 7.150 Å^(*b*)^		*c* = 7.150 Å^(*b*)^	

References: (*a*) Mumme *et al.* (1995 ▸), (*b*) Udagawa *et al.* (1980 ▸), (*c*) Smith *et al.* (1965 ▸), (*d*) Jost *et al.* (1977 ▸), (*e*) Midgley (1952 ▸), (*f*) Regourd *et al.* (1968 ▸), (*g*) Sarkar (1980 ▸), (*h*) Il’inets & Bikbau (1990 ▸), (*i*) Saalfeld (1975 ▸) and (*j*) Bredig (1950 ▸).

† The notation in parentheses refers to the extension of the respective unit cell with respect to the unit-cell parameters of 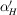 (of the adjusted setting) required to obtain the superstructure.

**Table 2:** Determined unit-cell parameters from all measured belite crystals in the different clinker samples and the already reported values

No. (polymorph)	*a* (Å)	*b* (Å)	*c* (Å)	α (°)	β (°)	γ (°)
clinker_0						
1 (β)	5.537	6.717	9.278	90.4	94.3	90.3
2 (β)	5.525	6.750	9.274	89.6	94.1	90.3
3 (β)†	5.497	6.780	9.246	90.3	94.3	90.0
4 ( 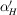 )	5.496	6.776	9.252	89.9	89.9	89.5
clinker_2.5						
1 (β)†	5.544	6.690	9.306	90.0	94.2	89.5
2 ( 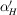 )	5.514	6.765	9.250	90.5	89.5	89.8
clinker_5						
1 (β)†	5.538	6.783	9.279	89.9	94.6	90.8
2 (β)	5.478	6.751	9.334	89.6	94.2	90.2
3 ( 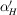 )	5.444	6.775	9.348	90.4	90.3	89.8
Literature						
β (Jost *et al.*, 1977 ▸)	5.502	6.745	9.297	90.0	94.59	90.0
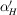 (Mumme *et al.*, 1995 ▸)	5.519	6.767	9.303	90.0	90.0	90.0

† Twinned crystal from which one of the unit cells is provided.

**Table 3:** Resulting figures of merit from the structure refinements of 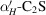 in space group *Pnma* from *JANA2006* The number of reflections, goodness of fit (GoF), *R* and *R*_w_ parameters are calculated and reported from observed and all (obs/all) reflections. The criterion for observed reflections was *I*(**g**) > 3σ(**g**). The ‘Refl./Param.’ measure refers to the number of observed reflections over the number of refined parameters. *R*_1_ and *R*_w_ are based on the square root of reflection intensities. Dynamical refinements were carried out with *N*_or_ of 128, *g*_max_ of 1.6 Å^−1^, 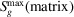 of 0.01 Å^−1^, 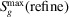 of 0.1 Å^−1^ and *RS_g_* of 0.4

	clinker_0		clinker_2.5	
	Kinematical	Dynamical	Kinematical	Dynamical
Completeness (%)	72.9		85.3	
*R*_int_	0.135		0.116	
No. of reflections at 0.7 Å	415/436	1710/2440	451/498	1489/1996
Refl./Param.	16.0	12.5	17.3	10.4
GoF	15.1/14.7	2.03/1.77	13.6/12.9	2.37/2.09
*R*	0.205/0.208	0.0557/0.0787	0.191/0.200	0.0550/0.0708
*R*_w_	0.251/0.251	0.0562/0.0585	0.226/0.226	0.0607/0.0623

**Table 4:** Superspace groups compatible with the *Pnma* space group and one modulation wavevector along the a* axis

Superspace group	Reflection conditions
*Pnma*(α00)000	*hk*0*m*: *h* = 2*n*, 0*kl*0: *k* + *l* = 2*n*
	*h*00*m*: *h* = 2*n*, 0*k*00: *k* = 2*n*, 00*l*0: *l* = 2*n*
*Pnma*(α00)0*s*0	*hk*0*m*: *h* = 2*n*, *h*0*lm*: *m* = 2*n*, 0*kl*0: *k* + *l* = 2*n*
	*h*00*m*: *h* + *m* = 2*n*, 0*k*00: *k* = 2*n*, 00 l0: *l* = 2*n*
*Pnma*(α00)00*s*	*hk*0*m*: *h* + *m* = 2*n*, 0*kl*0: *k* + *l* = 2*n*
	*h*00*m*: *h* + *m* = 2*n*, 0*k*00: *k* = 2*n*, 00*l*0: *l* = 2*n*
*Pnma*(α00)0*ss*	*hk*0*m*: *h* + *m* = 2*n*, *h*0*lm*: *m* = 2*n*, 0*kl*0: *k* + *l* = 2*n*
	*h*00*m*: *h* + *m* = 2*n*, 0*k*00: *k* = 2*n*, 00*l*0: *l* = 2*n*

**Table 5:** Resulting figures of merit (obs/all) from the dynamical refinements of 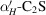 in *JANA2006* The criterion for observed (obs) reflections was *I*(**g**) > 3σ(**g**). The ‘Refl./Param.’ measure refers to the number of observed reflections over the number of refined parameters in the least-squares procedure. *R* and *R*_w_ are based on the square root of reflection intensities. *N*_or_ of 128, *g*_max_ of 1.6 Å^−1^, 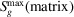 of 0.01 Å^−1^, 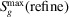 of 0.1 Å^−1^ were selected in both data sets, but *RS_g_* was set to 0.4 for clinker_0 and 0.5 for clinker_2.5.

	clinker_0	clinker_2.5
Main reflections		
No. of reflections at 0.7 Å	1726/2500	1848/2466
*R*	0.0710/0.0954	0.0763/0.0928
*R*_w_	0.0730/0.0757	0.0825/0.0841
Satellite reflections		
No. of reflections at 0.7 Å	2007/4796	1228/4531
*R*	0.199/0.279	0.159/0.312
*R*_w_ (%)	0.195/0.208	0.178/0.199
All reflections		
No. of reflections at 0.7 Å	3733/7296	3076/6997
Refl./Param.	29.6	23.8
*R*	0.123/0.182	0.0964/0.169
*R*_w_	0.112/0.120	0.0985/0.105

**Table 6:** Distances (Å) between split positions for the average structures and the total modulated displacements (Å) for the modulated structures of 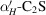

		Average structure		Modulated structure	
Atom	Mumme *et al.* (1995 ▸)	clinker_0	clinker_2.5	clinker_0	clinker_2.5
Ca1	0.34770			0.404 (1)	0.320 (1)
Ca2	0.24394			0.396 (1)	0.324 (1)
Si1				0.272 (1)	0.278 (1)
O1	0.58797†	0.696 (12)†	0.670 (11)†	0.938 (1)	0.81 (1)
O2	0.63470	0.684 (6)	0.22 (5)	0.665 (1)	0.617 (1)
O3	0.76495	0.790 (5)	0.754 (10)	0.797 (1)	0.723 (1)

† These values correspond to the O1—O1_2 distance.
