# Supplementary material for: Three-dimensional electron diffraction on clinkers: the belite α′ H incommensurate modulated structure
Source: Acta Crystallogr B Struct Sci Cryst Eng Mater. 2024 Mar 6;80(Pt 2):105–16. doi: 10.1107/S205252062400146X (PMC10994169; doi:10.1107/S205252062400146X)
Supplement: Supplementary file 4 [file b-80-00105-sup4.pdf]

STRUCTURAL SCIENCE
CRYSTAL ENGINEERING
MATERIALS

Volume 80 (2024)

Supporting information for article:

Three-dimensional electron diffraction on clinkers: the belite α_H' incommensurate modulated structure

Sergi Plana-Ruiz, Emilia Götz, Thomas Neumann, Peter Schwesig and Ute Kolb

STRUCTURAL SCIENCE
CRYSTAL ENGINEERING
MATERIALS

Volume 80 (2024)

Supporting information for article:

Three-dimensional electron diffraction on clinkers: the belite \alpha_H^' incommensurate modulated structure

Sergi Plana-Ruiz, Emilia Götz, Thomas Neumann, Peter Schwesig and Ute Kolb

Table S1 Acquisition and output parameters from the successful structure solutions of direct methods in Sir2014 for the analysed data sets. “clinker_0” data sets were acquired at 750 mm of camera length, while “clinker_2.5” and “clinker_5” data sets at 1 m. R_{int} is based on structure factors and R is based on structure factors and the BEA algorithm (Luca Cascarano *et al.*, 2010). “Ref.” stands for reference, “Refl.” for reflections and “Param.” for structure parameters.

	“clinker_0”				“clinker_2.5”		“clinker_5		
Ref. Crystal Number:	1 (β)	2 (β)	3 (β*)	4 (α'_H)	1 (β*)	2 (α'_H)	1 (β*)	2 (β)	3 (α'_H)
Tilt Range (º)	-45/45	-45/45	-45/45	-45/45	-34/45	-60/60	-45/45	-45/45	-45/45
Acq. Patterns (#)	91	91	91	91	81	121	91	91	91
CCD Exposure Time (s)	2	1	1	1	0.2	0.5	1	1	1
Num. of Reflections (#)	3671	3681	3632	3575	1376	1643	1559	1529	1536
Ind. Refl. at 0.7 Å (#)	696	825	865	444	550	512	616	601	424
Completeness (%)	65.9	78.1	81.9	72.9	52.1	85.3	58.6	57.0	70.8
Refl./Param. Ratio (-)	16.4	19.5	21.2	11.8	12.5	13.5	13.7	13.8	11.7
Overall B (Å^2)	1.01	0.95	0.90	1.84	1.04	1.97	0.64	0.73	2.01
R_{int} (%)	11.5	8.1	8.1	13.5	15.4	11.6	8.13	10.9	12.7
R (%)	17.0	20.0	23.3	14.8	23.6	16.0	26.5	17.7	20.8

* Twinned crystal from which one of the twin structure solutions is provided.

Table S2 Structural parameters of the incommensurately modulated model from the "clinker_0" diffraction data set. Unit-cell parameters obtained from eADT and scaled according to the scale factor of the β-C_2S data sets were used for the refinement. Values in parentheses for the different parameters are the estimated standard deviations. The dynamical refinement was carried out with N_{or} of 128, g_{max} of 1.6 Å^{-1}, $S_g^{max}(matrix)$ of 0.01 Å^{-1}, $S_g^{max}(matrix)$ of 0.1 Å^{-1} and RS_g of 0.4.

	**Ca1**	**Ca2**	**Si**	**O1**	**O2**	**O3**
x/a	0.16654(15)	0.50881(13)	0.28050(20)	0.72280(29)	0.49130(36)	0.15780(39)
y/b	0.75	0.75	0.25	0.48270(32)	0.81010(45)	0.32280(47)
z/c	0.42860(24)	0.70450(24)	0.58812(37)	0.82190(46)	0.45150(62)	0.43520(65)
Occup.	0.5	0.5	0.5	1.0	1.0	1.0
B_{iso} (Å^2)	1.03(3)	0.55(2)	0.79(3)	1.18(4)	1.53(6)	1.69(6)
			Harmonic Function Parameters			
$A_1(xsin)$	0	0	0	-0.02638(50)	-	-
$B_1(xcos)$	0	0	0	0.03824(48)	-	-
$A_2(ysin)$	-0.02368(32)	-0.03250(26)	-0.01781(42)	-0.02070(49)	-	-
$B_2(ycos)$	0.02811(30)	-0.01538(32)	-0.01703(43)	0.000491(48)	-	-
$A_3(zsin)$	0	0	0	0.00964(76)	-	-
$B_3(zcos)$	0	0	0	-0.03646(65)	-	-
			Crenel Function Parameters			
Δ	-	-	-	-	0.5	0.5
$x^0_{s,4}$	-	-	-	-	0.7150(22)	0.0025(18)

Table S3 Structural parameters of the incommensurately modulated model from the "clinker_2.5" diffraction data set. Unit-cell parameters obtained from eADT and scaled according to the scale factor of the β-C_2S data sets were used for the refinement. Values in parentheses for the different parameters are the estimated standard deviations. The dynamical refinement was carried out with N_{or} of 128, g_{max} of 1.6 Å^{-1}, $S_g^{max}(matrix)$ of 0.01 Å^{-1}, $S_g^{max}(matrix)$ of 0.1 Å^{-1} and RS_g of 0.5.

	**Ca1**	**Ca2**	**Si**	**O1**	**O2**	**O3**
x/a	0.16766(12)	0.50894(13)	0.28114(16)	0.72400(28)	0.49140(31)	0.15750(34)
y/b	0.75	0.75	0.25	0.48183(43)	0.80560(54)	0.31530(53)
z/c	0.42933(11)	0.70467(11)	0.58733(15)	0.82170(24)	0.45180(30)	0.44020(29)
Occup.	0.5	0.5	0.5	1.0	1.0	1.0
B_{iso} (Å^2)	1.33(2)	0.722(9)	0.80(3)	2.19(4)	2.13(5)	2.06(5)
			Harmonic Function Parameters			
$A_1(xsin)$	0	0	0	0.02570(44)	-	-
$B_1(xcos)$	0	0	0	-0.03327(40)	-	-
$A_2(ysin)$	-0.01738(37)	0.02735(30)	0.01406(49)	0.01938(63)	-	-
$B_2(ycos)$	-0.02311(33)	-0.01049(36)	0.02090(48)	-0.00247(64)	-	-
$A_3(zsin)$	0	0	0	0.01061(39)	-	-
$B_3(zcos)$	0	0	0	-0.02919(31)	-	-
			Crenel Function Parameters			
Δ	-	-	-	-	0.5	0.5
$x^0_{s,4}$	-	-	-	-	0.7772(24)	0.4957(20)

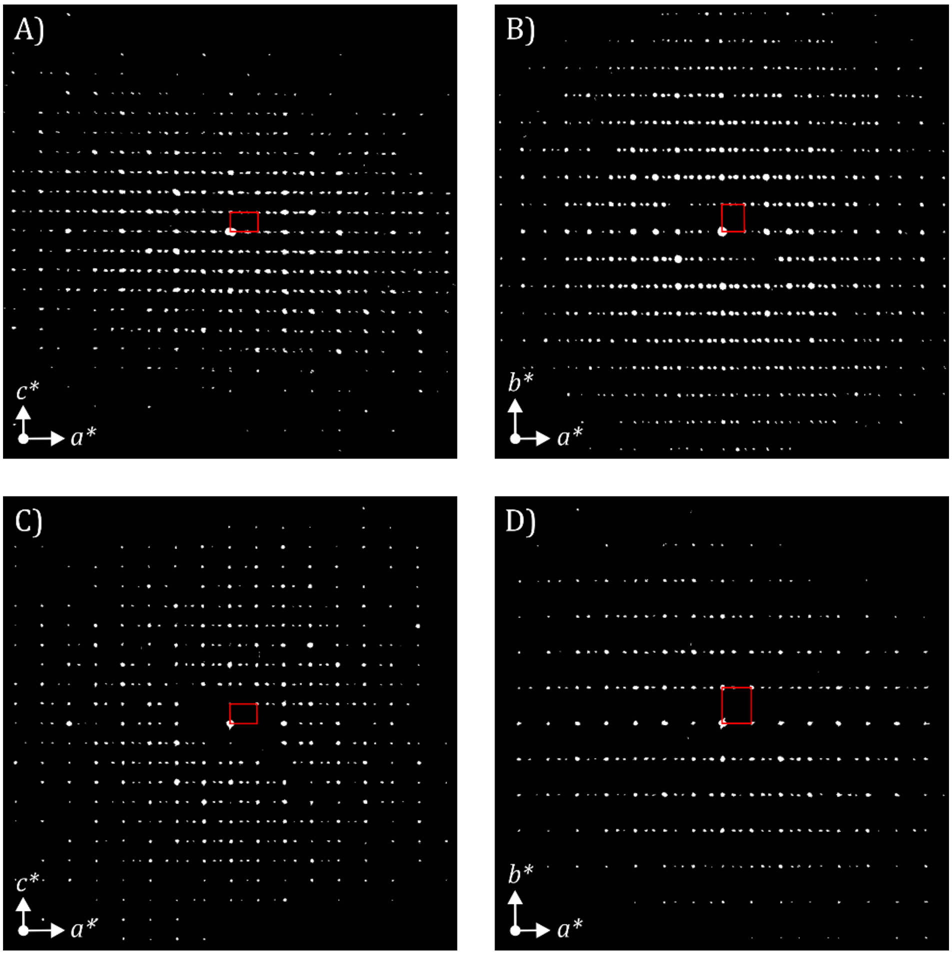

Figure S1 Projections along $b*$ and $c*$ axes of the reconstructed observable diffraction space for “clinker_0” (upper figures) and “clinker_2.5” (lower figures), respectively. The red rectangle in all figures represents the projected average unit cell along the corresponding axis.

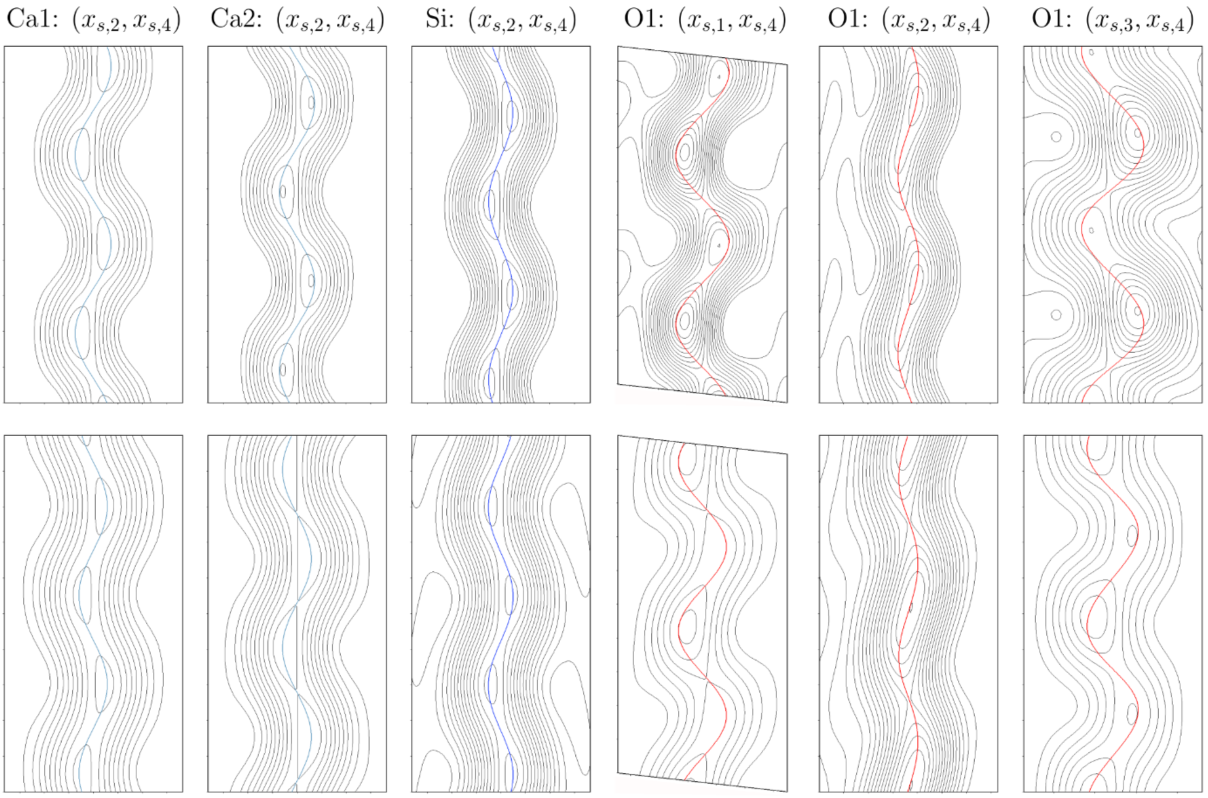

Figure S2 De Wolff sections for Ca1, Ca2, Si and O1 that show modulation. The coloured lines represent the harmonic functions obtained from the final dynamical refinement. Upper figures correspond to “clinker_0” and lower ones to “clinker_2.5”.
